# Early Outcomes Of Decentralized Care for Rifampicin-Resistant Tuberculosis in Johannesburg, South Africa: An Observational Cohort Study

**DOI:** 10.1371/journal.pone.0164974

**Published:** 2016-11-03

**Authors:** Rebecca Berhanu, Kathryn Schnippel, Erika Mohr, Kamban Hirasen, Denise Evans, Sydney Rosen, Ian Sanne

**Affiliations:** 1 Right to Care, Johannesburg, South Africa; 2 Health Economics and Epidemiology Research Office, Department of Internal Medicine, School of Clinical Medicine, Faculty of Health Sciences, University of the Witwatersrand, Johannesburg, South Africa; 3 Clinical HIV Research Unit, Department of Internal Medicine, School of Clinical Medicine, Faculty of Health Sciences, University of the Witwatersrand, Johannesburg, South Africa; 4 Médecins Sans Frontières, Khayelitsha, Cape Town, South Africa; 5 Center for Global Health & Development, Boston University, Boston, MA, United States of America; Rutgers Biomedical and Health Sciences, UNITED STATES

## Abstract

**Objective:**

We describe baseline characteristics, time to treatment initiation and interim patient outcomes at a decentralized, outpatient treatment site for rifampicin-resistant TB (RR-TB).

**Methods:**

Prospective observational cohort study of RR-TB patients from March 2013 until December 2014. Study subjects were followed until completion of the intensive phase of treatment (6 months), transfer out, or a final outcome (loss from treatment (LFT) or death).

**Results:**

214 patients with RR-TB were enrolled in the study. Xpert MTB/RIF was the diagnostic test of rifampicin resistance for 87% (n = 186), followed by direct PCR on AFB positive specimen in 14 (7%) and indirect PCR on cultured isolate in 5 (2%). Median time between sputum testing and treatment initiation was 10 days (IQR 6–21). Interim outcomes were available in 148 patients of whom 78% (n = 115) were still on treatment, 9% (n = 13) had died, and 14% (n = 20) were LFT. Amongst 131 patients with culture positive pulmonary TB, 85 (64.9%) were culture negative at 6 months, 12 were still sputum culture positive (9.2%) and 34 had no culture documented or contaminated culture (26%). Patients who initiated as outpatients within 1 week of sputum collection for diagnosis of RR-TB had a significantly lower incidence of LFT (IRR 0.30, 95% CI: 0.09–0.98). HIV co-infection occurred in 178 patients (83%) with a median CD4 count 88 cells/ml^3^ (IQR 27–218).

**Conclusions:**

Access to decentralized treatment coupled with the rapid diagnosis of RR-TB has resulted in short time to treatment initiation. Despite the lack of treatment delays, early treatment outcomes remain poor with high rates of death and loss from care.

## Introduction

Driven by an HIV prevalence exceeding 12% [1], tuberculosis (TB) is South Africa’s leading cause of death [2]. In 2014, nearly 19,000 cases of rifampicin-resistant TB (RR-TB) were diagnosed in South Africa making it second only to India in the absolute number of cases [3]. RR-TB, defined as resistance to rifampicin (RIF), encompasses rifampicin (RIF) mono-resistant TB, multi-drug resistant TB (MDR), and extensively drug-resistant TB (XDR).

Prior to 2011, all RR-TB in South Africa was treated in inpatient facilities for the duration of the intensive phase of treatment for six to eight months. A variety of barriers including diagnostic delays, inpatient bed shortages, and patient reluctance to be hospitalized resulted in long delays prior to treatment initiation [4]. Prior to 2011, the average time between sputum sampling and inpatient admission averaged 16 weeks in 2005–2006 in Kwa-Zulu Natal province (KZN) [5], 10 weeks in the Western Cape in 2007 [6], and 16 weeks in Northwest Province in 2009 [7]. These delays contributed to the extraordinarily high mortality rates with 40% of MDR-TB patients dying before treatment initiation in a study conducted in Tugela Ferry, South Africa between 2005 and 2007 [8]. For those who did initiate treatment, outcomes were poor with treatment success rates ranging between 46% and 54% [9] [10] [11].

In order to increase treatment facility capacity, minimize delays, and improve outcomes [12–14], in 2011 the South African National TB Programme announced a policy of “decentralized and deinstitutionalized” treatment for drug-resistant tuberculosis (DR-TB) [15,16] allowing treatment to be administered at the primary care level. Shortly after, Xpert MTB/RIF (Cepheid, Sunnyvale, CA, USA), a rapid molecular test which identifies both TB and rifampicin resistance, was endorsed as the first-line diagnostic test for TB suspects in 2012 [17].

We aim to describe outcomes of routinely delivered outpatient, decentralized care for RR-TB in the public sector. Using data from a public sector, hospital-based outpatient clinic in Johannesburg, South Africa we describe patient characteristics, time to treatment initiation, and early treatment outcomes.

## Methods

### Study Site

The study was conducted at the outpatient TB Focal Point clinic at Helen Joseph Hospital, a public academic hospital in Johannesburg, South Africa. The clinic is supported by a nongovernmental partner, Right to Care, and follows public sector diagnostic and treatment guidelines. The study site was one of three decentralized DR-TB treatment sites in Gauteng province, all of which were based at public hospitals. All patients diagnosed with DR-TB in the province were referred to one of these sites or to the established centralized, inpatient hospital.

**Fig 1:**
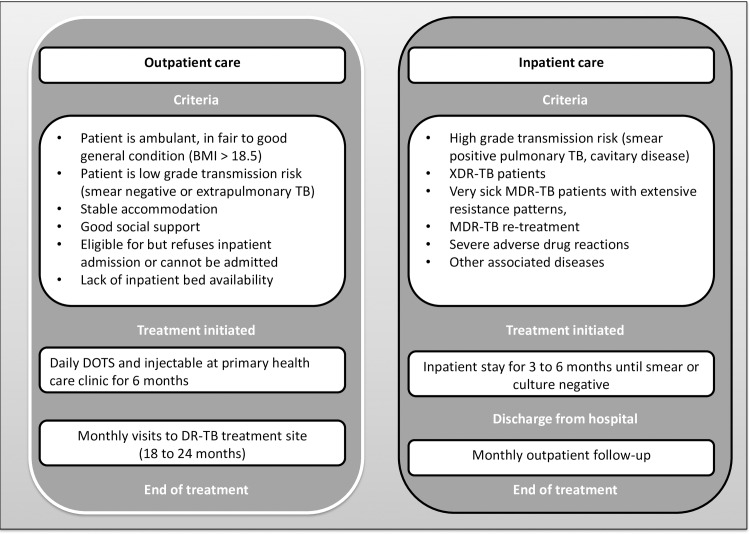
South African National Department of Health policy on eligibility for decentralized drug-resistant TB care.

Eligibility criteria for outpatient treatment have been established by the National Department of Health and take into account transmission risk as well as clinical and social criteria, as illustrated in Fig 1 [18]. Patients eligible for outpatient care should be ambulatory and AFB smear-negative.

The study clinic has a defined geographic catchment area from which patients are referred for care. Treatment for RR-TB is initiated at the first clinic visit. Concurrently with treatment initiation patients are counseled and tested for HIV if their status is unknown, sputum is obtained for drug-susceptibility testing, baseline blood tests including full blood count, kidney, liver and thyroid function testing are sent and the patient is scheduled for audiometry testing. Baseline CD4 count is sent in HIV-infected patients. Antiretroviral therapy (ART) has been integrated with RR-TB care; all TB patients are eligible for ART initiation regardless of CD4 count. The district TB coordinators are responsible for conducting home visits to ascertain infection control practices in the patient’s home and workplace and testing contacts. Following RR-TB treatment initiation, a follow-up visit is scheduled at two weeks, with monthly follow-up conducted thereafter for the 18–24 months of treatment. A month’s supply of medication is given at each visit; daily injectable drugs are administered at the patient’s primary health care clinic (PHC), which is typically a short distance from their home, during the intensive phase of therapy. Varying approaches to directly observed therapy (DOT) are implemented at PHCs, with some only providing the daily injectable treatment and allowing patients to self-administer oral medications for the intensive phase of therapy and others monitoring oral medication intake throughout treatment. Following treatment completion or cure, patients are followed up at six-monthly check-up visits for two years to screen for relapse.

### Drug susceptibility testing

Xpert MTB/RIF has been the first-line diagnostic tool used for suspected pulmonary TB (PTB) since mid-2012 in South Africa. Following diagnosis of RR-TB using Xpert MTB/RIF, sputum smears are examined using fluorescent microscopy (auramine). Liquid cultures are done in Mycobacterial Growth Indicator Tubes (MGIT) at the National Health Laboratory System’s (NHLS) central TB laboratory in Johannesburg. Molecular testing for resistance to isoniazid and rifampicin is done using line probe assay (LPA, GenoType MTBDR*plus*, HAIN). For patients with documented rifampicin resistance, phenotypic drug-sensitivity testing (DST) on liquid culture is done for isoniazid, ethambutol, streptomycin, kanamycin and ofloxacin. Phenotypic DST is no longer routinely repeated for rifampicin when resistance is detected by LPA unless specifically requested. LPA for second line drugs (GenoType MTBDR*sl*, HAIN) was not available during the study period.

### Standardized treatment regimens

The South African RR-TB guidelines stipulate that patients with RR-TB be treated with a standardized second-line regimen that includes the injectable agent kanamycin given intramuscularly for six to eight months (intensive phase of treatment) and four oral drugs, moxifloxacin, ethionamide, terizidone and pyrazinamide, given for 18 to 24 months [15]. In rifampicin mono-resistant TB cases where isoniazid susceptibility is confirmed by phenotypic DST, isoniazid is substituted for ethionamide. In patients diagnosed with inhA mutations high dose isoniazid (10 to 15mg/kg) and para-aminosalicylate sodium (PAS) are added to the regimen. In patients who develop hearing loss clinicians can chose to reduce the frequency or prematurely discontinue kanamycin treatment. Alternative drugs such as bedaquiline and linezolid for patients experiencing treatment toxicity became available in June 2015 and were not in use during the study period [19]. Patients with further resistance to aminoglycosides or fluoroquinolones (i.e. pre-XDR-TB and XDR-TB) are referred to the provincial inpatient TB hospital (transferred-out) for individualized treatment regimens.

### Study design

We conducted a prospective observational study of adult (≥18) patients who initiated treatment for RR-TB between March 2013 and December 2014 (22 months). This cohort included patients who had resistance to rifampicin detected by phenotypic or molecular tests, regardless of resistance to other drugs.

Study subjects were followed prospectively through medical record review until the earlier of either i) six months of treatment (end of the intensive phase); or ii) a final outcome was reached (loss from treatment (LFT) or death). Patients transferred to another treatment site (e.g. for inpatient care or to another province) or who had discordant drug-resistant test results during the intensive phase of treatment were excluded from the analysis of outcomes. Patients who were transferred in for care after receiving more than one month of treatment at another site were excluded from the study.

### Data collection and analysis

Following patient informed consent, clinical and laboratory data were prospectively entered into the study database. Information collected included demographic characteristics, previous history of TB infection and treatment, HIV status and ART, TB molecular and culture-based testing, laboratory data, adverse events, chest x-rays, and audiometry testing results. Adverse events were documented in patient files by clinical providers as part of routine care. Information about adverse events was extracted from patient files using a standardized case report form. All adverse events were graded as mild (grade 1), moderate (grade 2), severe (grade 3), potentially life threatening (grade 4), or fatal (grade 5) according to the AIDS Clinical Trials Group grading system [20].

Analysis was performed using Stata version (13.1) (StataCorp, College Station, TX USA). Descriptive statistics are presented using counts, proportions, and medians with interquartile range (IQR). We used Poisson regression with robust standard errors [21] to calculate the relative risk of dying during RR-TB treatment and of experiencing loss from treatment compared to remaining in care, by patient and clinical characteristics. All outcomes were evaluated at the same point in time (6 months) thus incidence rate ratios (IRR) are presented with 95% confidence intervals (95% CI). Characteristics identified *a priori* and analyzed were male sex, age category (0 to 29 years, 30 to 49 years, and 50 years and older), HIV infection, on ART at second-line TB treatment initiation, low CD4 count (≤ 100 cells/mm^3^), low body mass index (BMI <18.5), severe anemia (hemoglobin, Hb < 8.0 g/dL) compared to moderate (Hb 8.0–10.9 g/dL) or no anemia, treatment initiation site (outpatient facility compared to the hospital), time to treatment initiation (within one week of diagnosis), cavities present as per chest x-ray results, disease site (pulmonary TB versus extrapulmonary TB (EPTB)), smear status at treatment initiation (sputum smear positive versus sputum negative), baseline drug susceptibility pattern (rifampicin mono-resistant TB, MDR, and rifampicin resistant by Xpert MTB/RIF, and isoniazid mutations in patients with MDR (inhA vs katG). We present unadjusted results; cell counts were too small to present the adjusted models.

Ethical approval was granted by the Human Research Ethics Committee at the University of the Witwatersrand. Participants provided written informed consent to participate in this study. The study is presented according to the STROBE checklist for observational cohorts (http://www.strobe-statement.org/)

## Results

**Fig 2:**
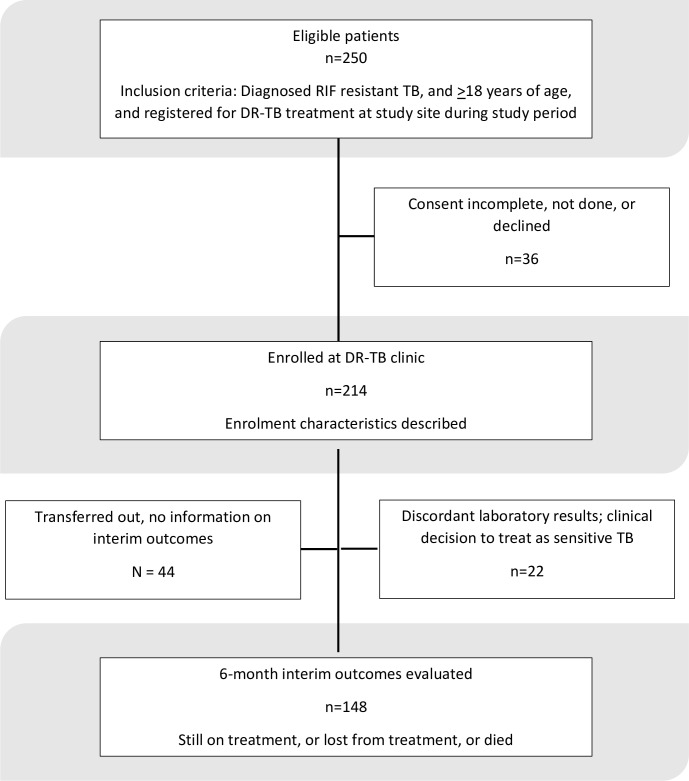
Description of cohort.

During the study period, 250 patients initiated treatment for RR-TB and met study eligibility criteria. Of these, 36 patients either declined participation, did not undergo informed consent, or had incomplete consent and were excluded, leaving 214 enrolled in the study (Fig 2).

The median age of enrolled study participants at diagnosis was 36 years (IQR 29–43) and 104 (49%) were male (Table 1). Median BMI at treatment initiation was 22.3 (IQR 20.1–24.6) among the 80% of patients with results available. Baseline median kidney function (glomerular filtration rate, GFR) was 65.5 ml/min/1.73m^2^ (IQR 57–78) with 43% of patients having mild kidney dysfunction (GFR 60–89) and 26% having moderate kidney dysfunction (GFR 30–59); 20% of patients were missing baseline GFR. Median baseline Hb (n = 159) was 11.0 g/dL (IQR 9.3–12.5) and 7.0% (n = 15) had severe anemia with Hb less than 8.0 g/dL.

**Table 1:** Descriptive characteristics at initiation of second-line TB treatment of RR-TB patients.

Characteristic	Count (n = 214)		Proportion %	IQR
**Patient characteristics**				
Male		104	48.6%	
Age		n = 214	(median) 36	29–43
	Age < 30	54	25.2%	
	Age 30 to 49	140	65.4%	
	Age 50+	20	9.3%	
Secondary schooling		184	86.0%	
Employed		88	41.1%	
South African		182	85.0%	
Children under 5 years at home		75	36.8%	
**Clinical conditions / status**				
Initiated treatment as inpatient		44	20.6%	
Pregnant	(n = 110)	6	5.5%	
Diabetes	(n = 214)	10	4.7%	
HIV-infected		178	83.2%	
	CD4 count, cells/mm^3^	n = 153	(median) 88	27–218
	Low CD4 count, CD4 < = 100	83	54.2%	
	On antiretroviral therapy at DR-TB treatment initiation	91	51.1%	
Body mass index (BMI) **kg**/m^2^		n = 172	(median) 22.3	20.1–24.6
	Low BMI, BMI <18.5	22	12.8%	
Glomerular filtration rate (GFR)*,ml/min/1.73m^2^		n = 182	(median) 65.5	57–78
	Mild kidney dysfunction GFR of 60–89	93	51.1%	
	Moderate kidney dysfunction GFR of 30–59	55	30.2%	
Hemoglobin, g/dL		n = 159	(median) 11.0	9.3–12.5
	Moderate anemia Hb of 8.0–10.9 g/dL	64	40.2%	
	Severe anemia Hb of <8.0 g/dL	15	9.4%	

* GFR calculated using Cockcroft-Gault formula where serum creatinine is measured in umol/L

HIV status was known for all patients; 178 (83%) were HIV infected with a median CD4 of 88 cells/mm^3^ (IQR 27–218 cells/ mm^3^). Of the HIV-infected patients, 91 (51%) were on ART at RR-TB treatment initiation. Diabetes was present in 10 (5%) of patients. Of the 110 female patients, 6 were pregnant at referral.

The majority of patients, 141 (66%), had no prior history of TB treatment; 62 (30%) had been previously treated with first line anti-TB drugs and 11 (5%) with second line anti-TB drugs TB (Table 2). EPTB occurred in 53 (25%) patients with TB lymphadenitis and disseminated TB being the most common forms; 10% of patients (n = 21) had only EPTB. Baseline chest x-ray results were available for 195 patients and revealed abnormalities consistent with tuberculosis in 168 (86%) and cavitary disease in 56 (33%).

**Table 2:** TB disease of RR-TB patients at enrolment.

Characteristic	Description	Count (n = 214)	Proportion
Presenting symptoms¶			
	Cough	159	74.3%
	Night sweats	128	59.8%
	Fever	125	58.4%
	Weight loss	109	50.0%
	Any one of cough, night sweats, fever, or weight loss	183	85.5%
Patient category			
	New	141	65.9%
	Previously treated, 1st line drugs	62	29.0%
	Previously treated, 2nd line drugs	11	5.1%
Pulmonary TB			
	PTB only	161	75.2%
	Both PTB and EPTB	32	15.0%
	EPTB only	21	9.8%
Extra pulmonary TB foci¶		n = 53	
	Lymph nodes	26	49.1%
	Pleural fluid	8	15.1%
	Meningitis	2	3.8%
	Disseminated	29	54.7%
	Abdominal	9	17.0%
Chest x-ray			
	Abnormal, suggestive of TB	168	78.5%
	Normal	27	12.6%
	Not done or report missing	19	8.9%
Chest x-ray findings		n = 195	
	Cavitary disease	56	28.7%
	Infiltrates	60	30.8%
	Pleural effusion or pleural fibrosis	34	17.4%
	Lymphadenopathy	39	20.0%

¶ Totals more than 100% as one patient could have more than one reported symptom or site of TB disease.

PTB: pulmonary tuberculosis; EPTB: extrapulmonary tuberculosis

Xpert MTB/RIF was the diagnostic test for rifampicin resistance in the majority of patients 87% (n = 186), followed by direct PCR on AFB positive specimen in 14 (7%) and indirect PCR on cultured isolate in 5 (2%) (Table 3). Amongst the 193 patients with PTB, positive sputum smear microscopy was present in 84 (44%) patients and positive sputum cultures occurred in 139 (72%) patients; in the remainder cultures were negative (n = 33), contaminated or not done (n = 21). Rifampicin mono-resistant TB was diagnosed in 89 (42%) patients, MDR-TB in 60 (28%) patients, XDR-TB in 4 (2%) patients and unconfirmed RR-TB diagnosed by Xpert MTB/RIF in 61 (29%) patients. Forty-four patients were diagnosed and initiated on second-line TB treatment while hospitalized. Amongst the 170 (79%) patients initiating RR-TB treatment as outpatients, median time from sputum collection to treatment initiation was 10 days (IQR 6–21), with 42% of patients initiating treatment within one week of sputum collection.

**Table 3:** TB disease of RR-TB patients at enrolment^, laboratory results.

Characteristic	Description	Count	Proportion
Sputum smear microscopy		n = 193	
	AFB positive (scanty or greater)	84	43.5%
	AFB negative	100	51.8%
	Not done (e.g. EPTB), missing	9	4.7%
Sputum culture		n = 193	
	Positive	139	72.0%
	Negative	33	17.1%
	Not done, contaminated, or missing	21	10.9%
DR-TB diagnosing test#		n = 214	
	Xpert MTB/RIF	186	86.9%
	PCR≠ direct on AFB+ sputum specimen	14	6.5%
	PCR≠ indirect on culture isolate	5	2.3%
	Could not be determined*	9	4.2%
DR-TB diagnosis		n = 214	
	RIF resistant by Xpert MTB/RIF, unconfirmed	61	28.5%
	RIF mono-resistant (INH sensitive)	89	41.6%
	MDR-TB (RIF and INH resistant)	60	28.0%
	XDR TB (MDR-TB plus resistance to a fluoroquinolone and 2nd line injectable)	4	1.9%
Days from sputum collection to treatment initiation ¶		n = 169	Median, IQR 10 (6–21)
	Initiated within one week	71	42.0%
	Initiated after one week	98	58.0%

^ Test results from +/- 3 months of treatment enrolment visit at DR-TB clinic; any positive test result in this period reported.

# Initial test indicating resistance to at least rifampicin. Tests for TB that do not indicate resistance or sensitivity to TB drugs (e.g. smear microscopy or culture) are excluded.

≠ PCR in use by the NHLS is GenotypeMTBDRplus

* More than one test results available prior to treatment start indicating rifampicin resistance

¶ Excludes patients initiating treatment in the hospital ward.

AFB: acid-fast bacillus; DR-TB: drug-resistant tuberculosis; INH: isoniazid; RIF: rifampicin; MDR-TB: multi-drug resistant TB; XDR TB: extensively drug resistant TB; IQR: inter-quartile range

Twenty-two (10%) patients with discordant laboratory results who were subsequently treated for drug-sensitive TB were excluded from the outcome analysis, as were 44 (21%) patients who transferred to other treatment facilities including 4 patients with XDR-TB who were transferred to the centralized hospital (Fig 2). Six-month interim outcomes were evaluated in the remaining 148 patients (Table 4). Thirteen patients (9%) died within 6 months of initiating RR-TB treatment, 20 (13%) were LFT, and 115 (78%) remained on treatment at the end of 6 months Amongst 131 patients with culture positive pulmonary TB at baseline, 85 (64.9%) were culture negative at 6 months, 12 were still sputum culture positive (9.2%) and 34 had no culture documented or contaminated culture (26%). Of the 130 HIV-infected patients included in the outcomes analysis, 122 (94%) were on ART during the study period. Adverse events (AEs) were reported in 70 (47%) patients, with a mean of 2.5 AEs per patient and at least one severe (grade 3 or 4) AE occurring in 46 (31%) of patients.

**Table 4:** Interim (6-month) RR-TB treatment outcomes.

Characteristic		Count	Proportion
No interim outcome		n = 66	
	Transfer out	44	66.7%
	Discordant results, treated as drug sensitive (discharged from RR-TB care)	22	33.3%
Interim (6-month) outcome		n = 148	
	On treatment, in care	115	77.7%
	Loss from treatment	20	13.5%
	Died	13	8.8%
Culture results at 6 months†		n = 131	
	Culture negative at 6 months	85	64.9%
	Culture positive at 6 months‡	12	9.2%
	Culture not documented or contaminated §	34	26.0%
On antiretroviral therapy		n = 130	
	On ART at RR-TB initiation	71	54.6
	Started on ART during RR-TB treatment	51	39.2%
	Not on ART at 6-month outcome	8	6.2%
Adverse events, by patient*		n = 148	
	No adverse event reported	78	52.7%
	One or more adverse event reported	70	47.3%
	Severe adverse event reported (grade 3 or 4)	46	31.1%

* Follow-up time for AE varied by patient’s time in care; thus patients remaining in care were more likely to have a AE reported because had more time ‘at risk’.

† Pulmonary TB patients who were culture positive at initiation

‡ Positive culture result more than 3 months after DR TB clinic enrolment, not followed by a negative culture result.

§ Cultures not done or contaminated

In univariate modified Poisson regression analysis (Table 5), patients with severe anemia had a 5-fold higher incidence of death during the first 6 months of treatment (IRR 5.16, 95%CI: 1.40–19.04). Patients who initiated as outpatients within 1 week of sputum collection for diagnosis of RR-TB had a significantly lower incidence of LFT within the first 6 months (IRR 0.30, 95% CI: 0.09–0.98). Men were also relatively less likely to be LFT compared to women although the difference was not statistically significant (IRR 0.42, 95% CI: 0.17–1.03). All deaths occurred among patients with PTB. HIV-infection (categorized according to CD4 count and ART status), sex, age category, previous history of TB, low BMI, and baseline drug-susceptibility pattern (rifampicin mono-resistant, rifampicin resistant by Xpert MTB/RIF, MDR) were not associated with an increase risk of death or loss during treatment.

**Table 5:** Relative risk* and 95% confidence intervals of dying or being lost from treatment.

		Relative risk of dying		Relative risk of loss from treatment	
		**IRR**	**95% CI**	**IRR**	**95% CI**
**Sex**	Male	1.14	0.40–3.23	0.42	0.17–1.03
**Age category**	Age 18–29	1.22	0.34–4.34	1.5	0.62–3.64
	Age 30–49	Reference		Reference	
	Age 50+	2.17	0.52–9.09	0.67	0.09–4.69
**HIV infection (ART)**	HIV-negative	Reference		Reference	
	HIV+ on ART	2.03	0.27–15.29	2.79	0.38–20.35
	HIV+ not on ART	1.22	0.14–10.31	2.44	0.32–13.35
**HIV infection (CD4)**	HIV-negative	Reference		Reference	
	HIV+ CD4>100	1	0.12–8.47	2.25	0.30–16.75
	HIV+ CD4≤ 100	2.48	0.33–18.67	3.10	0.42–22.78
**Body mass index**	Low BMI <18.5	1.03	0.13–8.10	0.48	0.07–3.43
**Anaemia**	No anaemia	Reference		Reference	
	Mild or moderate	1.28	0.34–4.85	0.71	0.26–1.98
	Severe	**5.16**	**1.40–19.04**	0.76	0.11–5.30
**Initiation model**	Inpatient	1.92	0.68–5.43	2.01	0.89–4.55
**Time to initiation**	≤ 1 week	1.04	0.24–4.47	**0.30**	**0.09–0.98**
**Pulmonary TB**	PTB site	All deaths PTB		0.52	0.20–1.38
**Sputum microscopy**	AFB+	2.21	0.67–7.27	0.63	0.22–1.81
**TB history**	Prior TB	0.67	0.22–2.09	1.01	0.44–2.32
**Kidney function**	Normal	Reference		Reference	
	Mild dysfunction	0.41	0.13–1.31	1.10	0.32–3.84
	Moderate dysfunction	0.31	0.06–1.46	1.53	0.42–5.58

* Relative risk calculated using Poisson regression with robust standard errors

Statistically significant differences bolded.

## Discussion

In a cohort of patients receiving treatment at one of South Africa’s first public sector outpatient clinics for RR-TB following the implementation of a national DR-TB management policy of decentralized treatment in 2011, we found that patients initiated treatment in a median of 10 days after sputum sampling. Six months after treatment initiation, three-quarters of patients remained in care; the remaining had died (9%) or experienced LFT (14%).

Reducing time to treatment initiation is critical to preventing ongoing transmission of disease [22–24] and reducing LFT. Our study found that patients who initiated therapy within 7 days of sputum collection where 70% less likely to be LFT at six months. The median delay of 10 days (IQR 6–21) in our study between sputum sampling and treatment initiation is significantly improved when compared to the situation prior to decentralization and the implementation of Xpert MTB/RIF when time to treatment initiation for RR-TB ranged between 49 days to 112 days [6,13,25] in South Africa. Our findings are similar to those from the community-based model of care being used in Khayelitsha, South Africa where the delay between sputum sampling and treatment decreased from a median of 71 days in 2006 to 8 days in 2013 following the implementation of Xpert MTB/RIF and the decentralization of treatment [26]. Because of the concurrent implementation of Xpert MTB/RIF and decentralization of RR-TB care in our setting it is not possible to determine the relative contribution of each to the reduction in time to treatment initiation seen in this study. Studies that have examined impact of decentralization and Xpert MTB/RIF separately found a reduction in time to treatment initiation from 50 days to 28 days after implementation of decentralization, and further reduction to 8 days after implementation of Xpert MTB/RIF [27]. Despite the reduced time to treatment initiation amongst outpatients, 21% of the patients in this cohort were diagnosed and initiated on treatment in the hospital, which suggests advanced disease at presentation and delayed diagnosis in a subset of patients.

Among HIV-infected patients the median CD4 count was 88 (IQR 27–218), suggesting that patients are presenting with advanced HIV disease. Fewer than half (44%) of those with pulmonary TB were smear positive at diagnosis. This highlights the importance of Xpert MTB/RIF for diagnosis of TB as the previous diagnostic algorithm that relied on AFB smear microscopy for the diagnosis of PTB would have missed these patients.

Our study demonstrates a high rate of LFT (14%) and death (9%) during the first 6 months of treatment. Studies conducted in Gauteng province prior to decentralization, during cohort years 2004–2010, demonstrated LFT rates ranging from 19.3–28.7% and rates of death between 15.4–21.8% with improved outcomes noted in later cohort years and, most notably, after implementation of ART for all DR-TB and HIV co-infected patients in 2010 [10,28]. Although there was a trend of increased death and LFT in our study among patients with HIV infection it did not achieve statistical significance. This is inline with other cohort studies that also found that RR-TB outcomes did not differ by HIV status in settings with access to ART [29,30]. The high rates of LFT in our cohort are similar to those in patients treated in a decentralized model in rural KZN, South Africa where rates of LFT were 15% at 6 months under decentralized care and 29% at the centralized hospital [14]. Rates of death in our study were lower than those observed in the same KZN cohort where 16% had died at 6 months [14]. Another study of decentralized care in Khayelitsha in the Western Cape found similarly high rates of LFT at the end of therapy of 29% [6]. The high rate of LFT across different cohorts and models of care attests to the need for shorter, better-tolerated treatment regimens, robust follow-up capacity within the framework of decentralized care and health-system strengthening [31]. In order to reduce LFT trained health outreach workers are needed to conduct contact tracing, provide home or workplace provision of DOT, and other patient support interventions [30,32,33]. As decentralization progresses, investment into community based follow-up and support systems should be prioritized.

Discordant rifampicin resistance results were common in our cohort and 10% of patients were found to have to have rifampicin discordance between Xpert MTB/RIF and subsequent LPA, phenotypic DST or repeat Xpert MTB/RIF. These discordant Xpert MTB/RIF results have been reported on previously and noted to be associated with delayed Xpert MTB/RIF probe hybridization [34]. Treatment for patients with discordance was individualized.

Our study has several limitations. The small size of the cohort does not allow definitive conclusions regarding factors that may contribute to patient treatment outcomes, particularly the high rate of LFT. A number of patients who initiated treatment died prior to consenting to study participation and were therefore excluded from analysis. This resulted in an underreporting of early deaths in this cohort. Despite these limitations, this study adds to the body of literature describing programmatic outcomes of decentralized RR- TB treatment in resource-limited settings.

In conclusion, the decentralization of RR-TB treatment coupled with the rollout of Xpert MTB/RIF in South Africa has fostered rapid diagnosis and treatment initiation for RR-TB. However, six-month treatment outcomes in this cohort remain poor, with the high rates of early death and LFT underscoring the urgent need for better-tolerated and shortened regimens coupled with strengthened patient support and follow-up systems.
